# Supplementary material for: TIGIT limits immune pathology during viral infections
Source: Nat Commun. 2020 Mar 9;11:1288. doi: 10.1038/s41467-020-15025-1 (PMC7062903; doi:10.1038/s41467-020-15025-1)
Supplement: Supplementary file 1 — Supplementary Information [file 41467_2020_15025_MOESM1_ESM.pdf]

SUPPLEMENTARY INFORMATION

TIGIT limits immune pathology during viral infections

Schorer et al.

[*] Corresponding author: nicole.joller@immunology.uzh.ch

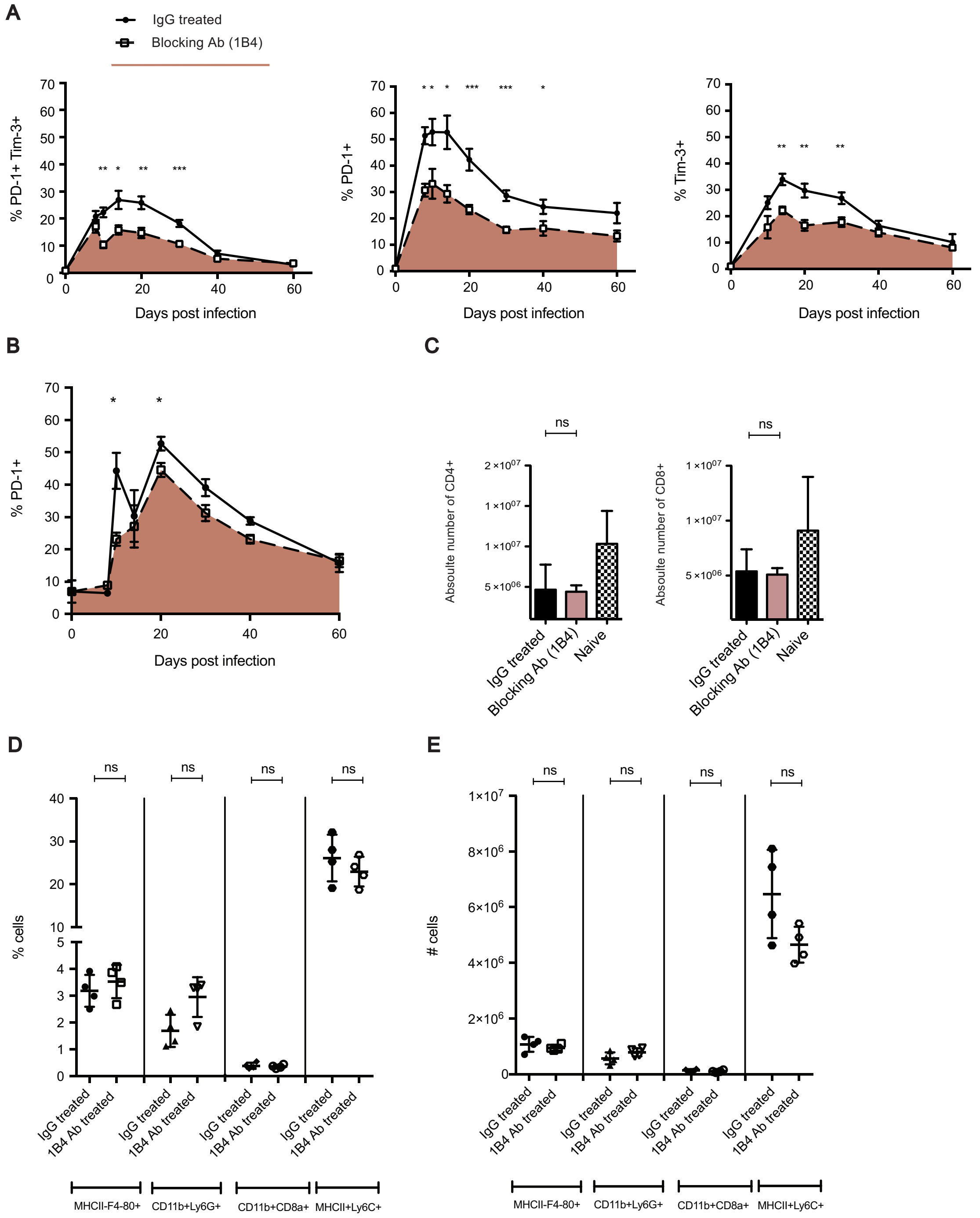

Supplementary Figure 1. Immune cell characterization during chronic LCMV infection +/- blocking anti-TIGIT (1B4) Ab treatment. C57BL/6 mice were infected with 2x10^{6} FFU LCMV clone 13 i.v. and treated with 100μg control IgG1 or anti-TIGIT Ab (1B4). (A) FACS quantification of PD-1 and Tim-3 expression on splenic CD8^{+} T cells throughout chronic LCMV infection is shown (n=23). (B) FACS quantification of PD-1 expression on splenic CD4^{+} T cells is shown, (n=12) (C) FACS quantification of CD4^{+} and CD8^{+} T cell counts in the spleen on day 30 p.i., (n=8). FACS quantification of (D) frequencies of myeloid cell populations and (E) absolute numbers of myeloid cell populations isolated from the spleen on day 5 p.i. (n=4). Each symbol in the scatter plot represents an individual mouse and bar graphs indicate the average value ± SEM. Statistical values. $p < 0.05$ (*), $p < 0.01$ (**), $p < 0.005$ (***) ns (not significant, $p > 0.05$) determined by Student's t test.

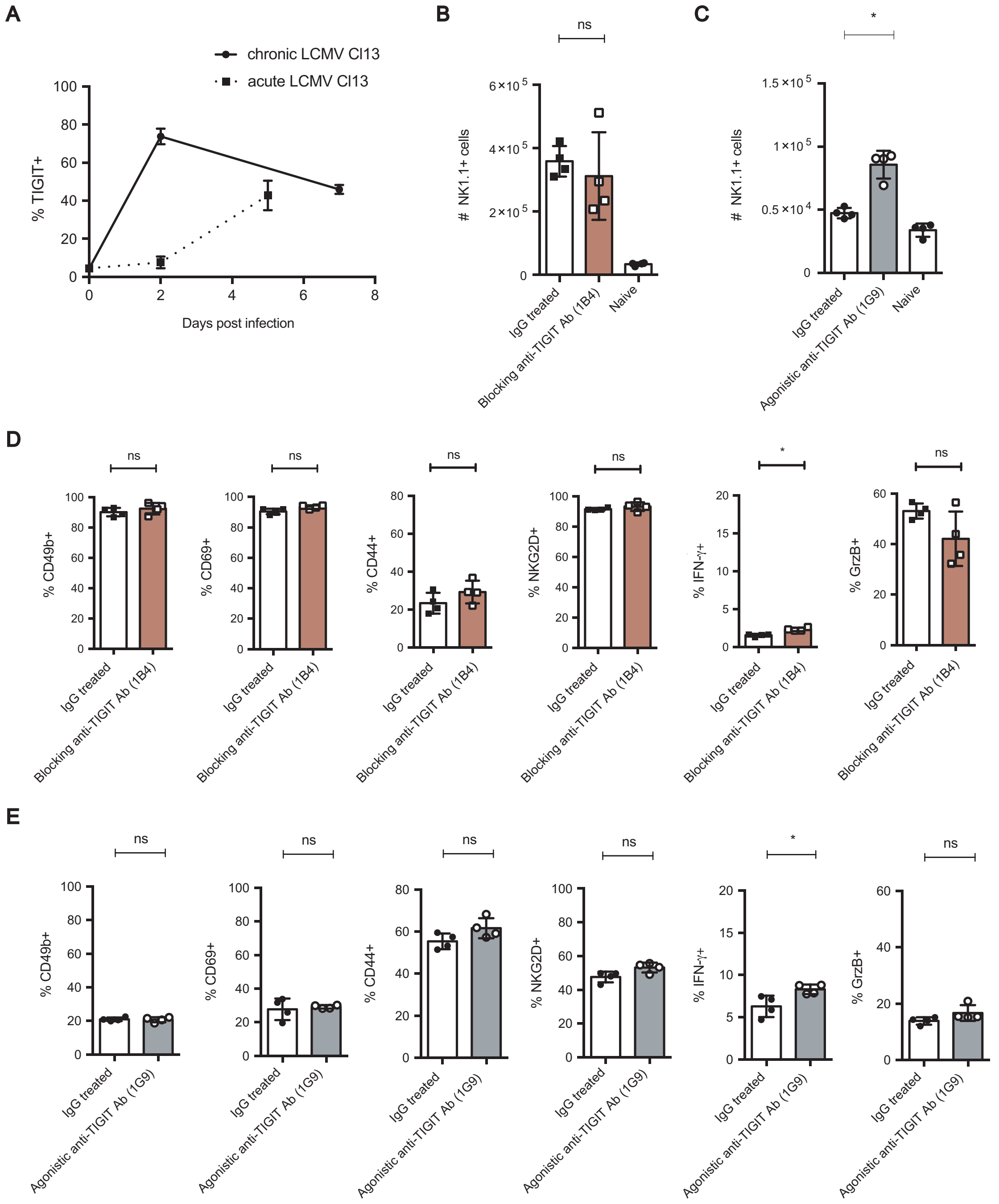

Supplementary Figure 2. NK cell phenotype during acute and chronic LCMV infection. C57BL/6 mice were infected with either $1x10^5$ FFU LCMV clone 13 i.v. (grey) or $2x10^6$ FFU LCMV clone 13 i.v. (red) and treated with 100µg control IgG1, agonistic anti-TIGIT Ab (1G9) and blocking anti-TIGIT Ab (1B4) on days 0, 2, 4 and 10 i.p. (A) TIGIT expression over the course of acute (dashed line) and chronic (solid line) LCMV infection measured on $NK1.1^+$ cells by FACS (n=4-5). (B) Absolute numbers of $NK1.1^+$ cells with and without blocking anti-TIGIT Ab (1B4) treatment on day 2 p.i. (n=4). (C) Absolute numbers of $NK1.1^+$ cells with and without agonistic anti-TIGIT Ab (1G9) treatment on day 2 p.i. (n=4). (D) Phenotypic and functional analyses of $NK1.1^+$ cells are shown on day 2 after chronic LCMV infection (n=4). (E) Phenotypic and functional analyses of $NK1.1^+$ cells are shown on day 5 p.i after acute LCMV infection, (n=4). Each symbol in the scatter plot represents an individual mouse and bar graphs indicate the mean value ± SD. Pooled data from 1-2 independent experiments are shown. Statistical values. $p < 0.05$ (*), $p < 0.01$ (**), $p < 0.005$ (***) ns (not significant, $p > 0.05$) determined by two tailed Mann-Whitney test.

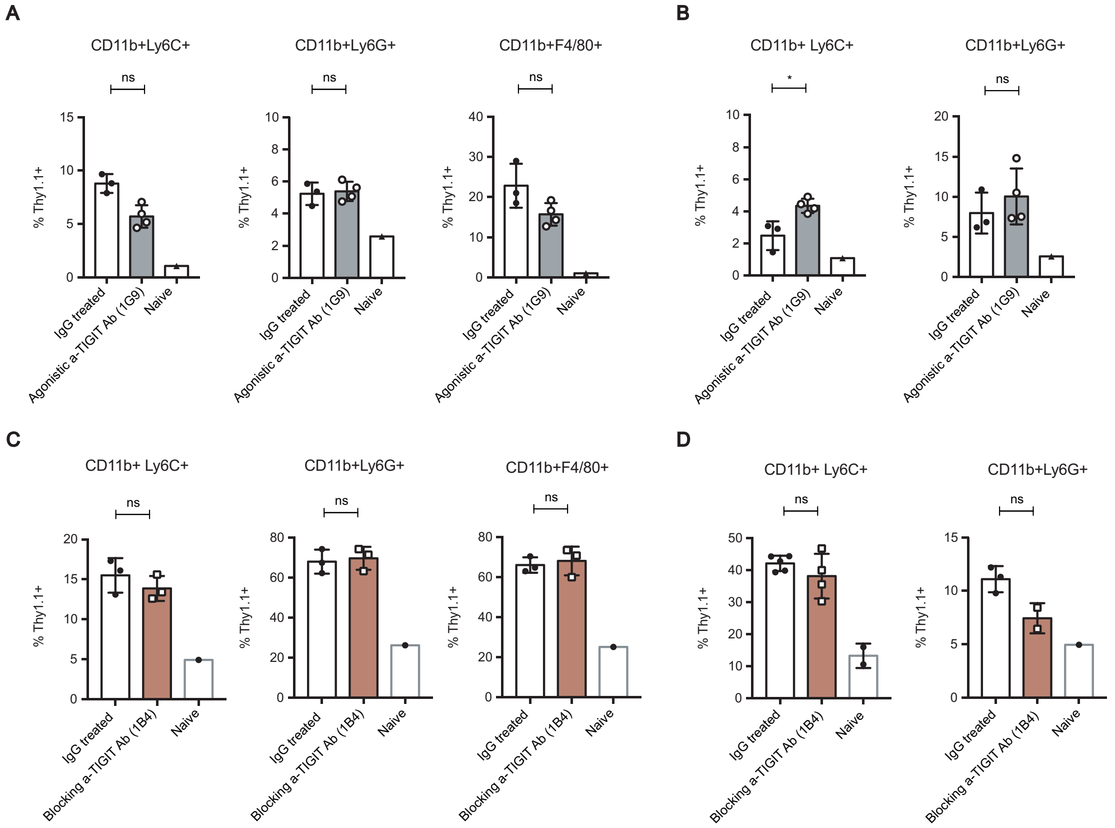

Supplementary Figure 3. Myeloid cell phenotype during acute and chronic LCMV infection. Thy1.1 IL-10 reporter mice were infected with either 1x10^5 FFU LCMV clone 13 i.v. (grey) or 2x10^6 FFU LCMV clone 13 i.v. (red) and treated with 100µg control IgG1, anti-TIGIT Ab (1G9) or anti-TIGIT (1B4) on days 0, 2, 4 and 10 i.p. (A) IL-10 Thy1.1 expression on splenic myeloid cell population on day 5 after acute infection is shown, (n=3-4), pooled data from 2 independent experiments. (B) IL-10-Thy1.1 expression on splenic myeloid cell population on day 14 after acute infection is shown, (n=3-4), pooled data from 2 independent experiments. (C) IL-10-Thy1.1 expression on splenic myeloid cell population on day 5 after chronic infection is shown, (n=3). (D) IL-10-Thy1.1 expression on splenic myeloid cell population on day 30 after chronic infection is shown, (n=2-5), pooled data from 1-2 independent experiments are shown. Each symbol in the scatter plot represents an individual mouse and bar graphs indicate the mean value ± SD. Statistical values. $p < 0.05$ (*), $p < 0.01$ (**), $p < 0.005$ (***) ns (not significant, $p > 0.05$) determined by two tailed Mann-Whitney test.

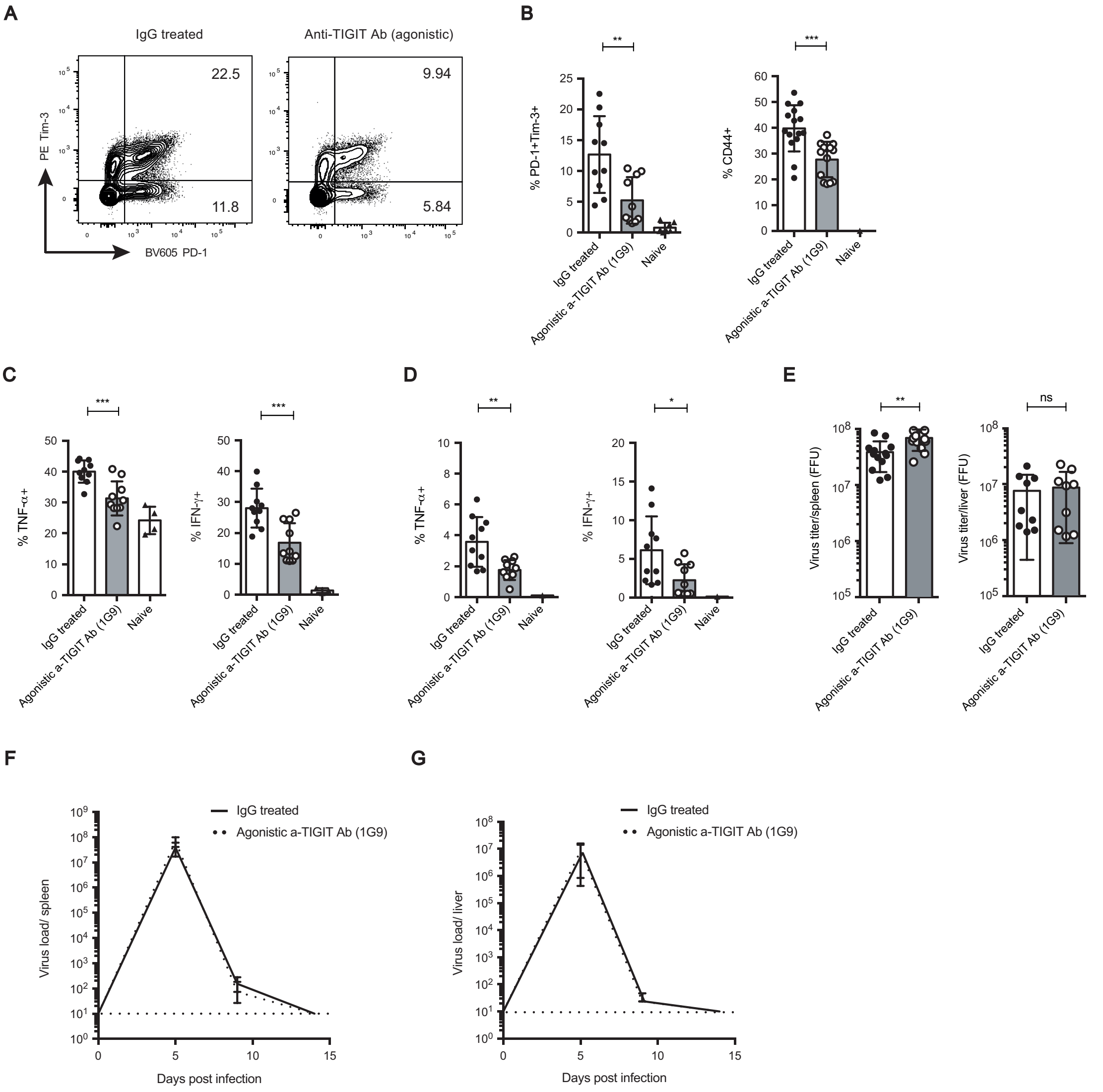

Supplementary Figure 4. TIGIT stimulation alters the T cell phenotype and cytokine profile of T cells without affecting virus control. C57BL/6 mice or Thy1.1 IL-10 reporter mice were infected with $1x10^5$ FFU LCMV clone 13 i.v. and treated with 100µg control IgG1 or anti-TIGIT Ab (1G9) on days 0, 2 and 4 i.p. (A) Representative FACS plots of co-inhibitory receptor expression on splenic $CD8^+$ T cells on day 5 p.i. are shown. (B) FACS quantification of co-inhibitory receptor expression and CD44 on splenic $CD8^+$ T cells on day 5 p.i., (n=10-15). FACS quantification of intracellular TNF-α and IFN-γ produced by (C) anti CD3 re-stimulated splenic $CD8^+$ T cells and (D) gp33 re-stimulated splenic $CD8^+$ T cells, (n=10). (E) Virus titers in the spleen and liver on day 5 p.i. are shown, (n=9-12). (F) Virus kinetic in the spleen (n=3) is shown. (G) Virus kinetic in the liver (n=3) is shown. Each symbol in the scatter plot represents an individual mouse and bar graphs indicate the average value ± SD. Pooled data from 2-3 independent experiments are shown. Statistical values. $p < 0.05$ (*), $p < 0.01$ (**), $p < 0.005$ (***) ns (not significant, $p > 0.05$) determined by Student's t test.

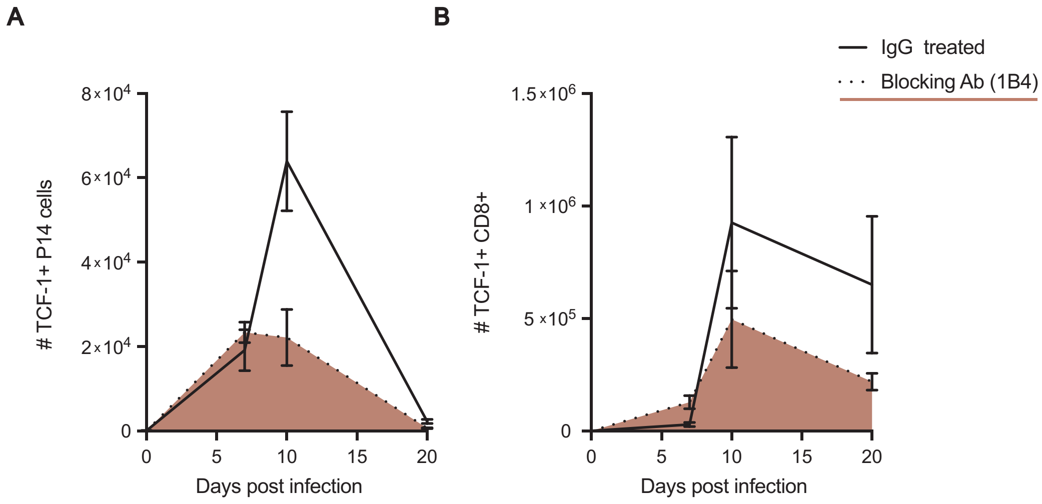

Supplementary Figure 5. TCF-1 expression on T cells during chronic LCMV infection. 10'000 P14 Ly5.1⁺ TCR transgenic CD8⁺ T cells specific for gp33 were transferred into C57BL/6 mice and infected with 2x10^6 PFU LCMV clone 13 i.v. and treated with 100μg control IgG1 or anti-TIGIT Ab (1B4) on days 0, 2, 4, 10 and 17 i.p. TCF-1 expression was measured on (A) P14 Ly5.1⁺ TCR transgenic CD8⁺ T cells and (B) endogenous CD8⁺ T cells on indicated days post infection in the spleen (n=2-7). Each symbol in the scatter plot represents an individual mouse and bar graphs indicate the mean value ± SEM. Pooled data of 2-3 independent experiments is shown.

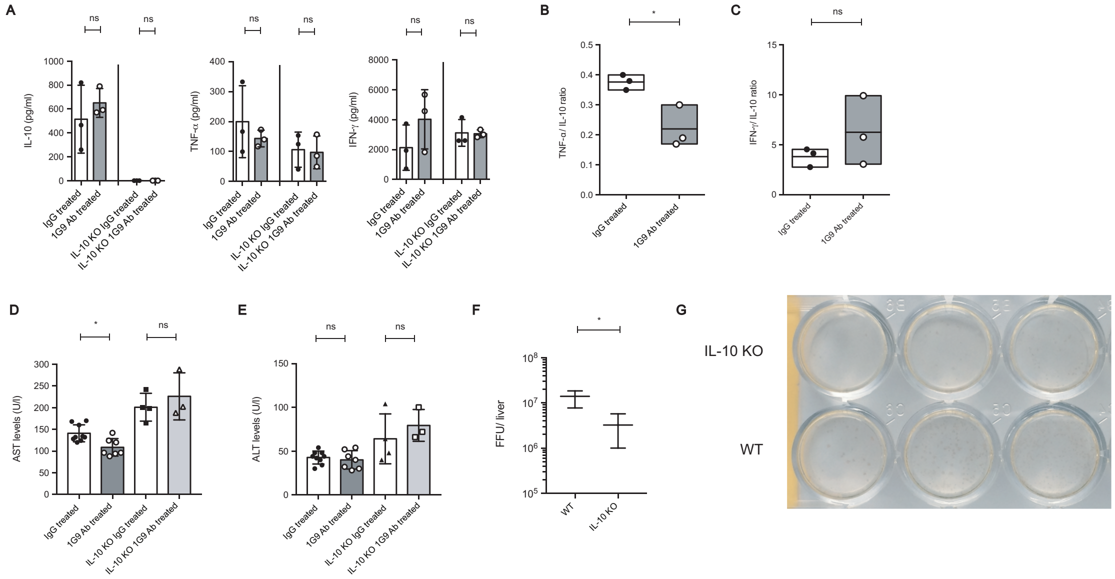

Supplementary Figure 6. Effect of TIGIT agonism in WT and IL-10 KO mice. C57BL/6 mice and IL-10 KO mice were infected with 1x10^5 PFU LCMV clone 13 i.v. and treated with 100μg control IgG1 or anti-TIGIT Ab (1G9) on days 0, 2 and 4 i.p. (A) Cytokine levels of C57BL/6 mice and IL-10 KO mice in *ex vivo* re-stimulated splenocyte supernatants on day 5 p.i. measured by flow cytometric bead assay (n=3). (B) The ratio of TNF-α to IL-10 protein in re-stimulated splenocyte supernatants on day 5 p.i. measured by flow cytometric bead assay (n=3). (C) The ratio of IFN-γ to IL-10 protein in re-stimulated splenocyte supernatants on day 5 p.i. measured by flow cytometric bead assay (n=3). Serum levels of (D) aspartate-aminotransferase (AST) and (E) alanine-aminotransferase (ALT) measured on day 5 p.i. are shown (n=3-9). (F)-Virus loads in WT and IL-10 KO mice on day 5 p.i. are shown (n=3). (G) Representative focus forming assay plate of WT and iL-10 KO mice on day 5 p.i. is shown. Each symbol in the scatter plot represents an individual mouse and bar graphs indicate the mean value ± SD. Pooled data of 2 independent experiments is shown. Statistical values. $p < 0.05$ (*), $p < 0.01$ (**), $p < 0.005$ (***) ns (not significant, $p > 0.05$) determined by one-way ANOVA (Holm-Sidak post test) if more than two groups were compared and one tailed Mann Whitney test if two groups were compared.

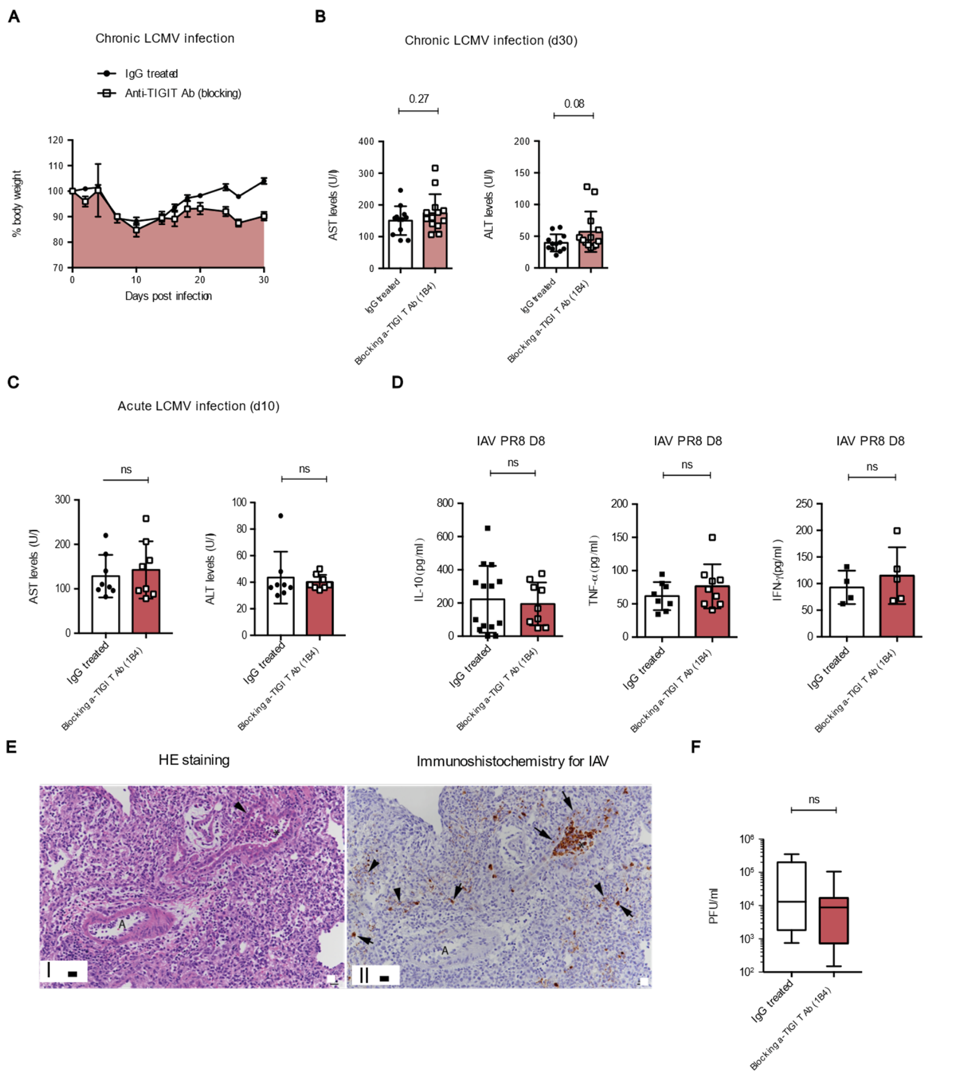

Supplementary Figure 7. Effect of blocking anti-TIGIT (1B4) Ab treatment in various infection models. C57BL/6 mice were infected with either 1x10^5 FFU LCMV clone 13 i.v., 2x10^6 FFU LCMV clone 13 i.v. or 200 PFU IAV PR8 i.n. and treated with 100μg control IgG1 or anti-TIGIT (1B4) on days 0, 2, 4, 10, 17, 24 i.p. (chronic dose, light red) or on days 0, 2, 4 (acute dose, dark red). (A) Weight loss curve of chronically infected mice treated with control IgG1 and anti-TIGIT (1B4) Ab is shown (n=10). (B) Serum levels of aspartate-aminotransferase (AST) and alanine-aminotransferase (ALT) measured on day 30 p.i. are shown (n=11-13). Serum levels of (C) aspartate-aminotransferase (AST) and alanine-aminotransferase (ALT) measured on day 10 p.i. are shown (n=8). (D) Cytokine levels in whole lung homogenates measured on day 8 p.i. IAV PR8 i.n. measured by flow cytometric bead assay are shown (n=4-15). **(E)** Histological features and IAV antigen expression in the lungs of or anti-TIGIT (1B4) Ab treated animals. (I) Consolidated area with increased interstitial cellularity and bronchiole (*) with degenerate epithelial cells (arrowhead) and some debris in the lumen. Artery (A) with activated endothelial cells (bulging into the vessel lumen). (II) Consecutive section showing IAV antigen expression in respiratory epithelial cells (arrows) and luminal cell debris in bronchiole (*), and in type I pneumocytes (arrowheads) and type II pneumocytes/alveolar macrophages (short arrows). (a) (HE stain), (b) (immunohistology for IAV antigen), Bars = 20 μm. (F) Virus load in lungs measured on day 8 p.i. by plaque assay (n=4). Each symbol in the scatter plot represents an individual mouse and bar graphs indicate the mean value ± SD. Statistical values. $p < 0.05$ (*), $p < 0.01$ (**), $p < 0.005$ (***) ns (not significant, $p > 0.05$) determined by Student's t test.

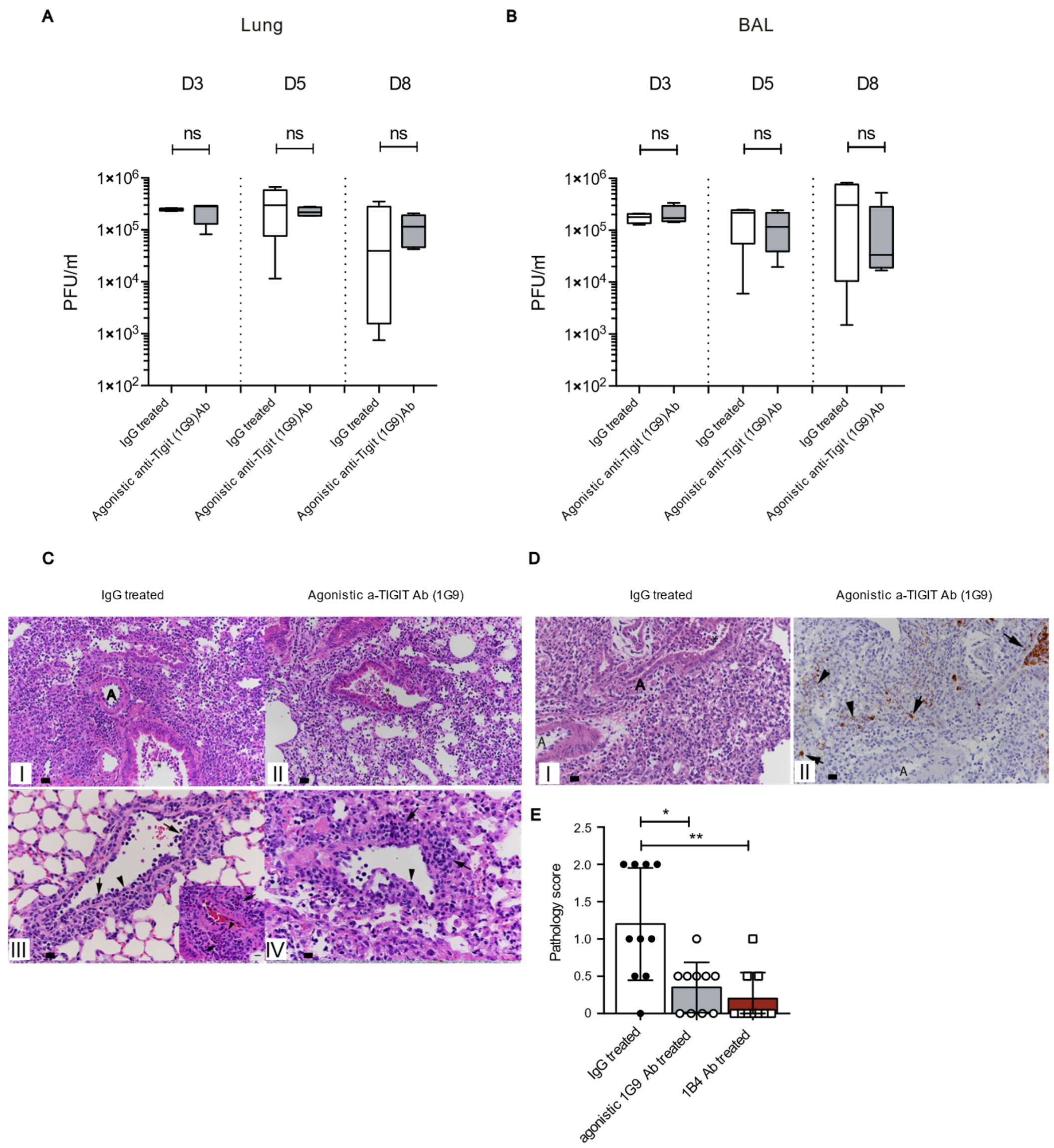

Supplementary Figure 8. Lung phenotype and virus quantification after influenza infection. C57BL/6 mice were infected i.n. with 200 PFU influenza A virus PR8 and treated with 100μg control IgG1 or anti-TIGIT Ab (1G9) on days 0, 2 and 4 i.p. Virus burden was measured by plaque assay on indicated days post infection (A) lung tissue and (B) bronchoalveolar lavage (n=4-5). (C) Histological features and (D) IAV antigen expression in the lungs on day 8 p.i.: Figure panel of IgG1 treated mice (C, I) Consolidated area with increased interstitial cellularity and bronchioles (*) with individual degenerate epithelial cells (arrowheads) and some debris in the lumen. Artery (A) with activated endothelial cells (bulging into the vessel lumen). (D, I) Consecutive section showing IAV antigen expression in respiratory epithelial cells (arrows) and luminal cell debris in bronchioles (*), and in type I pneumocytes (arrowheads) and type II pneumocytes/alveolar macrophages (short arrows). (C, III) Larger vein with activated endothelial cells (arrowhead) and rolling and emigrating leukocytes (arrows). Inset: Mild arteritis with neutrophils in the media (arrowhead) and as aggregates (arrows) in the adjacent tissue: Mice treated with agonistic anti-TIGIT (1G9) Ab (C, II): Moderately consolidated area with increased cellularity and bronchiole (*) with focal area of epithelial cell loss (arrow) and degenerate epithelial cells (arrowhead). (D, II) Section showing IAV antigen expression in respiratory epithelial cells (arrow) and luminal cell debris in a bronchiole (*), and in type I pneumocytes (arrowhead) and type II pneumocytes/alveolar macrophages (short arrows). (C, IV) Larger vein with activated endothelial cells (arrowhead) and mild perivascular mononuclear (macrophages and lymphocytes) infiltrates (arrows). (C) (HE staining), (D) (immunohistology for IAV antigen). Bars = 20 μm (C-D, I, II) and 10 μm (C, III, IV). (E) Pathology score of IgG1, agonistic anti-TIGIT (1G9) Ab or blocking anti-TIGIT (1B4) Ab treated lungs, single-blinded analysis. The degree of neutrophil infiltration and vasculitis was assessed by light microscopy. Box plot and whiskers displaying the minimum and maximum of all data points. Statistical values. $p < 0.05$ (*), $p < 0.01$ (**), $p < 0.005$ (***) ns (not significant, $p > 0.05$) determined by two tailed Mann-Whitney test (A-B) and Kruskal-Wallis test (E).

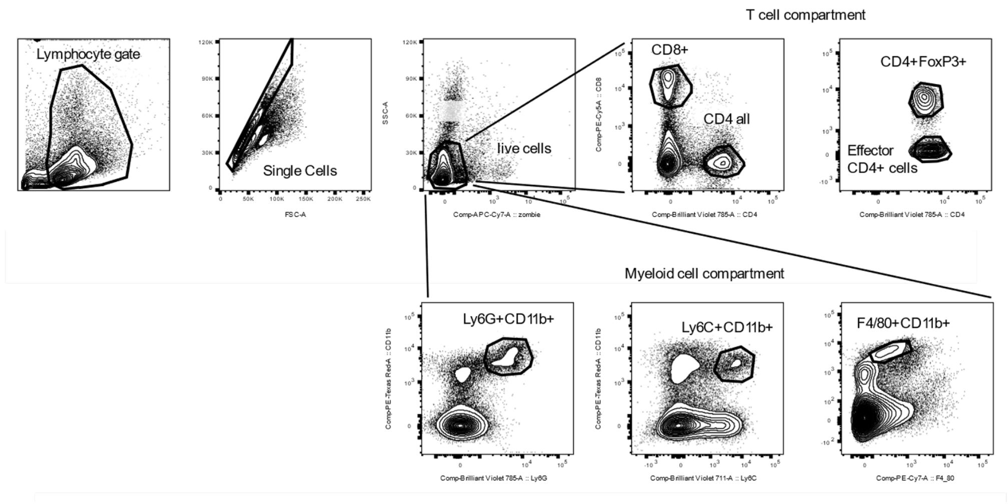

Supplementary Figure 9. Gating strategies. T cell compartment and myeloid immune cells.

Ab	Clone	Cat. number	Dilution	Supplier
CD4	RM4-5	100552	1:200	Biolegend
CD4	GK1.5	100453	1:200	Biolegend
PD-1	29F.1A12	135220	1:200	Biolegend
CD44	IM7	103026	1:400	Biolegend
CD8	53-6.7	100710	1:500	Biolegend
IFN-γ	XMG1.2	505808	1:300	Biolegend
Lag-3	C9B7W	125221	1:200	Biolegend
TIGIT	1G9	142110	1:50	Biolegend
TNF-α	MP6-XT22	506324	1:500	Biolegend
CD45.1	A20	110706	1:200	Biolegend
CD90.1	OX-7	202529/<br>202537	1:400	Biolegend
Granzyme B	GB11	MHGB05	1:50	Thermo Fisher
CD49b	DX5	108920	1:200	Biolegend
CD69	H1.2F3	104514	1:200	Biolegend
NKG2D	CX5	130208	1:100	Biolegend
CD11b	M1/70	101212	1.400	Biolegend
MHCII	M5/114.15.2	107604	1:800	Biolegend
Ly6C	HK1.4	128037	1:400	Biolegend
Ly6G	1A8	127645	1:400	Biolegend
NK1.1	PK136	108738	1:100	Biolegend
F4/80	BM8	123114	1:400	Biolegend
TCF1/TCF7	C63D9	2203S	1:100	Cell Signaling Technology
Tim-3	215008	FAB1529P	1:200	R&D Systems

Supplementary Table 1: FACS antibodies. Information about the antibodies used for FACS stainings is summarized.
