# Supplementary material for: TIGIT limits immune pathology during viral infections
Source: Nat Commun. 2020 Mar 9;11:1288. doi: 10.1038/s41467-020-15025-1 (PMC7062903; doi:10.1038/s41467-020-15025-1)
Supplement: Supplementary file 3 — Reporting Summary [file 41467_2020_15025_MOESM3_ESM.pdf]

nature research

Corresponding author(s): Nicole Joller

Last updated by author(s): Jan 27, 2020

Reporting Summary

Nature Research wishes to improve the reproducibility of the work that we publish. This form provides structure for consistency and transparency in reporting. For further information on Nature Research policies, see Authors & Referees and the Editorial Policy Checklist .

Statistics

For all statistical analyses, confirm that the following items are present in the figure legend, table legend, main text, or Methods section.

n/a	Confirmed	
☐	☒	The exact sample size (*n*) for each experimental group/condition, given as a discrete number and unit of measurement
☐	☒	A statement on whether measurements were taken from distinct samples or whether the same sample was measured repeatedly
☐	☒	The statistical test(s) used AND whether they are one- or two-sided<br>*Only common tests should be described solely by name; describe more complex techniques in the Methods section.*
☒	☐	A description of all covariates tested
☐	☒	A description of any assumptions or corrections, such as tests of normality and adjustment for multiple comparisons
☐	☒	A full description of the statistical parameters including central tendency (e.g. means) or other basic estimates (e.g. regression coefficient) AND variation (e.g. standard deviation) or associated estimates of uncertainty (e.g. confidence intervals)
☐	☒	For null hypothesis testing, the test statistic (e.g. *F*, *t*, *r*) with confidence intervals, effect sizes, degrees of freedom and *P* value noted<br>*Give P values as exact values whenever suitable.*
☒	☐	For Bayesian analysis, information on the choice of priors and Markov chain Monte Carlo settings
☒	☐	For hierarchical and complex designs, identification of the appropriate level for tests and full reporting of outcomes
☒	☐	Estimates of effect sizes (e.g. Cohen's *d*, Pearson's *r*), indicating how they were calculated

Our web collection on statistics for biologists contains articles on many of the points above.

Software and code

Policy information about availability of computer code

Data collection	FACS Diva
Data analysis	FlowJo 10, GraphPad Prism 6

For manuscripts utilizing custom algorithms or software that are central to the research but not yet described in published literature, software must be made available to editors/reviewers. We strongly encourage code deposition in a community repository (e.g. GitHub). See the Nature Research guidelines for submitting code & software for further information.

Data

Policy information about availability of data

All manuscripts must include a data availability statement. This statement should provide the following information, where applicable:

- Accession codes, unique identifiers, or web links for publicly available datasets
- A list of figures that have associated raw data
- A description of any restrictions on data availability

The data that support the findings of this study are available from the corresponding author upon reasonable request.

Field-specific reporting

Please select the one below that is the best fit for your research. If you are not sure, read the appropriate sections before making your selection.

☒ Life sciences ☐ Behavioural & social sciences ☐ Ecological, evolutionary & environmental sciences

For a reference copy of the document with all sections, see nature.com/documents/nr-reporting-summary-flat.pdf

Life sciences study design

All studies must disclose on these points even when the disclosure is negative.

Sample size	We assumed an average effect size of 30-50%, and a difference of 10-15% as biologically significant. The desired power was 90%, the significance level was set to $p<0.05\%$, the variance was 2.5 for systemic infections and 4.5 for intranasal infections (based on experience). Experimental groups were compared to control groups so a two sided test is used. Using the Pwr package in R this gave us a group size of 2.1-2.7 or 3.2 - 5.4, respectively, and we thus generally used groups of at least 3 animals for systemic infections and at least 5 animals for intranasal infections.
Data exclusions	No data was excluded from the analyses. Individual biological experiments were not included in the analyses when both the cytokine data and pathology data indicated that animals were not infected.
Replication	All the reported findings were replicated at least once. It is stated in all figure legends that each experiment was repeated at least once.
Randomization	Experimental groups were not randomized, but age-matched and housed in the same animal house for the duration of the experiment.
Blinding	All histo-pathological data was single-blinded for the analysis.

Reporting for specific materials, systems and methods

We require information from authors about some types of materials, experimental systems and methods used in many studies. Here, indicate whether each material, system or method listed is relevant to your study. If you are not sure if a list item applies to your research, read the appropriate section before selecting a response.

Materials & experimental systems

n/a	Involved in the study	
☐	☒	Antibodies
☒	☐	Eukaryotic cell lines
☒	☐	Palaeontology
☐	☒	Animals and other organisms
☒	☐	Human research participants
☒	☐	Clinical data

Methods

n/a	Involved in the study	
☒	☐	ChIP-seq
☐	☒	Flow cytometry
☒	☐	MRI-based neuroimaging

Antibodies

Antibodies used	A detailed antibody list is provided in Supplementary Table 1 of the manuscript
Validation	Antibodies were titrated in-house and used at the indicated dilation factors in Supplementary Table 1.

Animals and other organisms

Policy information about studies involving animals; ARRIVE guidelines recommended for reporting animal research

Laboratory animals	The study involved C57BL/6 (B6), Ly5.1, Thy1.1, Foxp3-GFP.KI and Thy1.1 IL-10 reporter, P14, Smarta, IL-10-/- and PKOB mice. Mice were age and gender matched within experiments and used at 8-16 weeks of age.
Wild animals	none
Field-collected samples	none
Ethics oversight	All experiments were performed in accordance with institutional policies and national regulations and have been reviewed and approved by the Cantonal veterinary office of Zurich.

Note that full information on the approval of the study protocol must also be provided in the manuscript.

Flow Cytometry

Plots

Confirm that:

- [x] The axis labels state the marker and fluorochrome used (e.g. CD4-FITC).
- [x] The axis scales are clearly visible. Include numbers along axes only for bottom left plot of group (a 'group' is an analysis of identical markers).
- [x] All plots are contour plots with outliers or pseudocolor plots.
- [x] A numerical value for number of cells or percentage (with statistics) is provided.

Methodology

Sample preparation	FACS stainings were performed on single-cell suspensions from spleen, liver and lung. The spleen samples were prepared by mechanical disruption in RPMI 1640 medium supplemented with 10% FCS, penicillin (100IU/ml) and 1% L-glutamine. Liver and lung were enzymatically digested for 30 minutes and immune cells isolated using a 30% Percoll (GE Healthcare) gradient. Red blood cells were removed by adding ACK lysis buffer (155mM NH4Cl, 10mM KHCO3, 0.1mM Na2EDTA, pH: 7.4) for 3 minutes. Cells were re-stimulated with either anti-CD3 (1µg/ml), gp33 peptide or gp61 peptide (EMC microcellections) and Brefeldin A solution (1x) at 37°C in 10% CO2 for 3-4h before staining. For surface stainings, antibodies were incubated for 20-30 minutes at RT in PBS. For intracellular cytokine staining, spenocytes were permeabilized using the Cytofix/Cytoperm kit (BD Biosciences) for 5 minutes at RT, followed by antibody incubation for 20-30 minutes at RT. For staining of transcription factors, cells were stained and permeabilized with the Foxp3/Transcription factor staining buffer set (eBioscience).
Instrument	BD LSR Fortessa or BD FACS CantoII analyzer (BD Bioscience)
Software	Data were acquired with Diva Software (BD Bioscience) and analyzed with FolwJo (TreeStar).
Cell population abundance	No sorted samples were used in the present study.
Gating strategy	The relevant gating strategies are depicted in Supplementary Figure 9. Gates were set based on samples stained with isotype control antibodies.

- [x] Tick this box to confirm that a figure exemplifying the gating strategy is provided in the Supplementary Information.
